# Tuning Ag/Co Metal Ion Composition to Control In Situ Nanoparticle Formation, Photochemical Behavior, and Magnetic–Dielectric Properties of UV–Cured Epoxy Diacrylate Nanocomposites

**DOI:** 10.3390/nano16020143

**Published:** 2026-01-21

**Authors:** Gonul S. Batibay, Sureyya Aydin Yuksel, Meral Aydin, Nergis Arsu

**Affiliations:** 1 Department of Chemistry, Yıldız Technical University, Istanbul 34220, Turkey; 2 Research Laboratories Application and Research Center (ALUAM), Turkish-German University, Istanbul 34820, Turkey; 3 Department of Physics, Yıldız Technical University, Istanbul 34220, Turkey

**Keywords:** photopolymerization, in situ nanoparticle synthesis, hybrid Ag/Co_3_O_4_ nanoparticles, photocuring kinetics, dielectric properties

## Abstract

In this study, we report a reproducible in situ photochemical method for the simultaneous synthesis of metallic and hybrid metal/metal oxide nanoparticles (NPs) within a UV–curable polymer matrix. A series of epoxy diacrylate-based formulations (BEA) was prepared, consisting of Epoxy diacrylate, Di(Ethylene glycol)ethyl ether acrylate (DEGEEA), and Phenylbis (2,4,6-trimethylbenzoyl) phosphine oxide (BAPO), which served as a Type I photoinitiator. These formulations were designed to enable the simultaneous photopolymerization and photoreduction of metal precursors at various Ag^+^/Co^2+^ ratios, resulting in nanocomposites containing in situ-formed Ag NPs, cobalt oxide NPs, and hybrid Ag–Co_3_O_4_ nanostructures. The photochemical, magnetic, and dielectric properties of the resulting nanocomposites were evaluated in comparison with those of the pure polymer using UV–Vis and Fourier Transform Infrared Spectroscopy (FT-IR), Photo-Differential Scanning Calorimetry (Photo-DSC), Thermogravimetric Analysis (TGA), Scanning Electron Microscopy (SEM), X-Ray Diffraction (XRD), Impedance Analysis, and Vibrating Sample Magnetometry (VSM). Photo-DSC studies revealed that the highest conversion values were obtained for the BEA-Ag_1_Co_1_, BEA-Co, and BEA-Ag_1_Co_2_ samples, demonstrating that the presence of Co_3_O_4_ NPs enhances polymerization efficiency because of cobalt species participating in redox-assisted radical generation under UV irradiation, increasing the number of initiating radicals and leading to faster curing and higher final conversion. On the other hand, the Ag NPs, due to the SPR band formation at around 400 nm, compete with photoinitiator absorbance and result in a gradual decrease in conversion values. Crystal structures of the NPs were confirmed by XRD analyses. The dielectric and magnetic characteristics of the nanocomposites suggest potential applicability in energy-storage systems, electromagnetic interference mitigation, radar-absorbing materials, and related multifunctional electronic applications.

## 1. Introduction

Photochemical synthesis of polymer nanocomposites is a unique strategy for nanoparticle (NP) and polymer network formation with in situ generation of metal or metal oxide NPs under mild and highly controllable conditions. Although there are several other synthesis methods, such as thermal, biochemical, and chemical reduction methods, the photochemical synthesis method provides rapid curing, spatial and temporal control of radical concentrations, and nanoscale confinement that allows precise regulation of nanoparticle growth [1,2,3,4]. Such advantages have positioned photochemically engineered nanocomposites as key materials for optical coatings, flexible electronics, sensing, high-k dielectrics, and electromagnetic wave attenuation technologies [5,6,7].

Irgacure 2959, Irgacure 651, and BAPO are well-known examples of Type I photoinitiators. Type II and one-component Type II photoinitiators are also used for the efficient in situ preparation of metallic and bimetallic NPs within a polymer matrix by forming initiating and photo-reducing radical species [8,9,10,11,12,13]. Upon UV exposure, Type-I photoinitiators undergo α-cleavage, ultimately producing highly reactive radical species that are capable of initiating photopolymerization and also reducing metal ions [14,15,16]. According to the literature, phosphine oxide-based photoinitiators produce radicals with strong reducing capability, and they exhibit high photobleaching efficiency, which leads to deeper curing and enhanced kinetics in multifunctional monomer systems [17,18,19]. Reported studies also indicate that the photoreduction of metal ions in acrylate-based matrices is strongly influenced by the photoinitiator structure, absorption characteristics, and the evolving polymer microenvironment [20,21,22,23,24].

Increasing viscosity causes restriction of diffusion during the photopolymerization reactions, which, on the other hand, facilitates the stabilization of early metal nuclei and promotes uniform nanoparticle size distributions. Transition metals such as cobalt undergo photoinduced ligand-to-metal charge transfer (LMCT) that generates auxiliary radicals for further accelerating polymerization kinetics [25,26]. Co NPs containing formulations, which are stated in the literature, often exhibit higher polymerization rates and conversions than Ag-rich systems [27,28].

The strong surface plasmon resonance (SPR) band of Ag NPs at around 400 nm, which partially overlaps with the photoinitiator absorption, results in reduced effective radical generation and slowing polymerization efficiency, as widely reported in plasmonic–photopolymer systems [29].

In situ formation of Ag, Co, and bimetallic NPs formed within photocured polymer networks during in situ photopolymerization typically results in crystalline metallic phases confined within an amorphous matrix, as confirmed by XRD, FT-IR, and SEM analyses. Nanoconfinement prevents uncontrolled growth of particles and contributes to obtaining enhanced optical, dielectric, and magnetic properties. It has been shown that the photocured hybrid organic–inorganic polymeric systems effectively stabilize metal NPs, improving transparency, dielectric behavior, and interfacial compatibility [30,31]. Hybrid Ag/Co_3_O_4_ nanostructures also exhibit synergistic charge mobility, enhanced interfacial polarization, and strong electromagnetic loss characteristics [32,33,34].

Nanocomposite materials containing metallic NPs have shown significant potential for the tunable permittivity, enhanced interfacial polarization, and strong Maxwell–Wagner–Sillars relaxation, which makes these materials suitable for advanced dielectric components [35,36,37,38,39,40]. NPs exhibiting magnetic properties, such as Co_3_O_4_, Fe_3_O_4_, and related spinels, introduce magnetic resonance and eddy current loss mechanisms that are crucial for electromagnetic interference (EMI) shielding applications and radar absorbing materials (RAMs) [41,42,43,44]. Hybrid systems consisting of both conductive Ag NPs and magnetic Co NPs are especially effective in broad-band wave absorption [45] due to the improved impedance matching, dual-loss mechanisms, and optimized microstructure control capability offered by photochemical curing.

The present study investigates epoxy diacrylate-based (BEA) photocurable formulations, which are designed to simultaneously undergo photopolymerization and in situ photoreduction of Ag^+^ and Co^2+^ ions under UV light using BAPO as a Type-I photoinitiator. This strategy enables us to control the in situ generation of Ag, Co, and Ag/Co NPs with various stoichiometric ratios. Additionally, photopolymerization kinetics were followed by photo-DSC studies, and the results demonstrated that the formulations, which consist of in situ formed Co NPs, exhibited accelerated polymerization and higher final conversions due to redox-assisted radical generation, whereas Ag-rich systems showed less curing efficiency due to SPR-induced competition. Detailed characterization of nanocomposites using UV–Vis, FT-IR, SEM, XRD, impedance spectroscopy, and VSM shows that the nanocomposites possess enhanced dielectric performance, magnetic responsiveness, and interfacial polarization behavior. These synergistic properties highlight the strong potential of the developed nanocomposites for applications in energy-storage devices and lightweight radar-absorbing materials.

## 2. Experimental

### 2.1. Chemicals

Phenylbis(2,4,6-trimethylbenzoyl)phosphine oxide (BAPO, Sigma-Aldrich) (Missouri, USA), Epoxy diacrylate (EA)) (Covestro, Germany), Di(Ethylene glycol)ethyl ether acrylate (DEGEEA, Aldrich) (Missouri, USA), Cobalt(II) chloride (CoCl_2_, anhydrous, Merck) (Darmstadt, Germany), Silver nitrate (AgNO_3_, Merck) (Darmstadt, Germany), and *N*,*N*′-Dimethylformamide (DMF, Merck) (Darmstadt, Germany) were used as received without any purification process.

### 2.2. Instruments

The analyses were performed using the following instruments. Varian Cary Win UV 50 Conc UV–Visible Spectrophotometer (Agilent, Santa Clara, CA, USA), Agilent Cary 630 FT-IR Spectrophotometer (Agilent, Santa Clara, CA, USA), Bandelin Sonopuls HD 2070 Homogenizer, Primarc UV Technology Mini UV–Curing Unit (Primarc, UK), Manual Roller Coater (40 μm thickness) (RK Print Coat Instrument, UK), SII6000 Exstar TG/DTA 6300 (Seiko Instruments, Chiba, Japan), Netzsch STA 449 F3 Jupiter Simultaneous Thermal Analyzer (STA–TG/DSC) (Selb, Germany), TA Instruments Q100 Photo-Differential Scanning Calorimeter (I = 30 mW cm^−2^) (New Castle, PA, USA), Malvern PANalytical X’Pert PRO X-Ray Diffractometer (Malvern, UK), Malvern PANalytical Empyrean MultiCore X-ray Diffractometer (Malvern, UK), MicroSense easyVSM Device (Lowell, MA, USA), FE-SEM (Thermo Fisher Quattro ESEM (Waltham, MA, USA) and HP 4192A Impedance Analyzer (operated at room temperature, 10–10^7^ Hz) (Yokogawa-Hewlett Packard Ltd., Tokyo, Japan).

### 2.3. Methods

#### 2.3.1. Photochemical Preparation of NPs in Solution

All solutions were prepared using the same photoinitiator (BAPO) concentration of 1 × 10^−3^ mol L^−1^. Formulations contained either AgNO_3_ or CoCl_2_ at a concentration of 1 × 10^−2^ mol L^−1^, while hybrid systems were prepared with AgNO_3_/CoCl_2_ molar ratios of 1:1, 1:2, and 2:1 by appropriately adjusting the precursor concentrations. After preparation, the solutions were irradiated for 30 min using a medium-pressure mercury lamp. Time-dependent UV–Vis absorption spectra were recorded simultaneously and are presented in Figure S1a–f of the Supporting Information.

#### 2.3.2. Preparation of Nanocomposite Materials via the UV–Curing Technique

Initially, 1 μL of *N*,*N*’-Dimethylformamide (DMF) was added to the weighed metal salts, and the mixtures were stirred with a magnetic stirrer for 24 h. Then, the photoinitiator Phenylbis(2,4,6-trimethylbenzoyl)phosphine oxide (BAPO) was added to the mixture, and the solutions were stirred for some extra minutes to obtain homogeneity. Once complete homogeneity was achieved, Epoxy diacrylate (EA) and Di(ethylene glycol) ethyl ether acrylate (DEGEEA) were added according to the formulation compositions. To ensure homogeneity, the formulations were subjected to ultrasonic homogenization at 20% power for 1 min while being kept in an ice bath. The sample coded BEA was prepared as a blank formulation without the addition of any metal salts. In this way, the influence of different metallic and metal/metal oxide NPs on the material properties could be systematically investigated. The formulations were prepared according to the weight ratios specified in Table S1, as provided in the Supporting Information.

Nanocomposite films with thicknesses ranging from 1 millimeter to several millimeters were prepared in silicone molds in the form of disks or rectangular prisms. Additionally, thin films of approximately 40 μm in thickness were prepared on aluminum and glass plates using a manual roller coater. The coated or molded formulations were cured using a mini-UV–curing device equipped with a 100 W medium-pressure mercury lamp, with a total of 100 passes. In this UV–curing system, 1 “pass” corresponds to 2 s of irradiation time.

#### 2.3.3. Measurement of Absorbance and Reflectance by UV–Visible and Fourier Transform Infrared (FT-IR) Spectroscopy

The absorbance spectra were recorded in the 200–800 nm wavelength range using a UV–Vis spectrophotometer and in the 650–4000 cm^−1^ wavenumber range using an FT-IR spectrophotometer. After the formulations were coated onto glass substrates as thin films (40 μm), they were cured for 100 s using a mini-UV–curing unit, and the absorption spectra were recorded before and after curing. The reflectance spectrum was recorded after 100 passes of irradiation (200 s). However, no noticeable changes were observed.

#### 2.3.4. Investigation of Photopolymerization Kinetics by Photo-DSC

The photopolymerization kinetics of formulations were investigated by Photo-DSC, with the photoinitiator content in the formulations given in Table S1, and each sample (1–3 mg) was weighed into aluminum pans and placed in the DSC unit. Measurements were carried out under isothermal conditions against a reference (empty) pan for an irradiation period of 600 s, under an inert N_2_ atmosphere with a flow rate of 50 mL min^−1^. The light intensity was measured as 30 mW cm^−2^ in the wavelength range of 220–400 nm. The heat flow was monitored as a function of reaction time under isothermal conditions. Both the polymerization rate and the conversion % were calculated as functions of time. The theoretical heat of reaction for acrylate double bonds was taken as $\Delta H_{p}^{theo}$ = 86 kJ mol^−1^ [46]. The polymerization rate (R_p_) was calculated using the following Equation (1):

(1) $$R_{p}=\frac{Q∕sM}{n\Delta H_{p}m}$$

where Q/s is the heat flow per second, M is the molar mass of the monomer, n is the number of double bonds per monomer molecule, and m is the mass of the monomer in the sample. The number of unsaturated bonds was taken as two for EA and one for DEGEEA. The variations in heat flow, polymerization rate, and conversion % with time were plotted accordingly.

#### 2.3.5. Investigation of the Thermal Properties of the Nanocomposite Materials

Samples (1–3 mg) were cut from the nanocomposite materials prepared in silicone molds. STA–TG/DSC measurements were performed under an inert N_2_ atmosphere in the temperature range of 30–800 °C, using a heating rate of 10 °C min^−1^.

#### 2.3.6. Investigation of the Surface Properties and Crystal Structures of the Nanocomposite Materials

For XRD analysis, the nanocomposite films coated on glass surfaces were peeled off and ground into powder using a mortar prior to measurement. The surfaces of the nanocomposite films prepared by coating onto metal substrates were examined using SEM.

#### 2.3.7. Investigation of the Dielectric Properties of Nanocomposite Materials

To determine the dielectric properties of the EA-based materials, cross-sections were taken from the nanocomposite samples prepared in silicone molds. The samples were assembled in a parallel-plate capacitor configuration between two metal electrodes. Measurements were carried out at room temperature using an impedance analyzer in the frequency range of 10 to 10^7^ Hz.

#### 2.3.8. Investigation of the Magnetic Properties of Nanocomposite Materials

The nanocomposite materials prepared in silicone molds were cut into cubic pieces with a mass of approximately 20 mg. The samples were then placed in a 5 mm quartz transverse sample holder for bulk analysis.

## 3. Results and Discussion

### 3.1. Effect of Ag/Co NPs Composition on the Optical Properties of UV–Cured Nanocoatings

**Figure 1:**
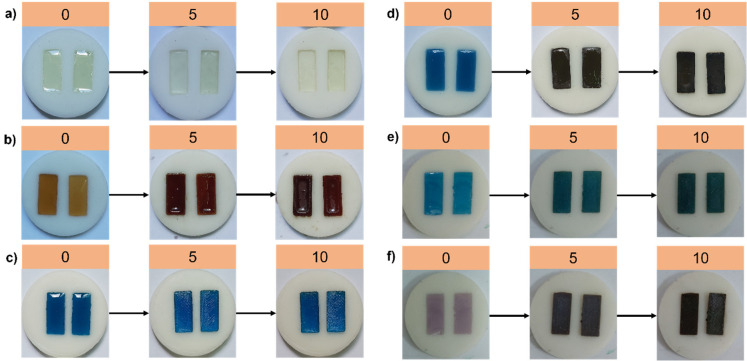
Changes observed during the curing process of EA/DEGEEA-based nanocomposite films within silicone molds: (**a**) BEA, (**b**) BEA-Ag, (**c**) BEA-Co, (**d**) BEA-Ag_1_Co_1_, (**e**) BEA-Ag_1_Co_2_, (**f**) BEA-Ag_2_Co_1_.

**Figure 2:**
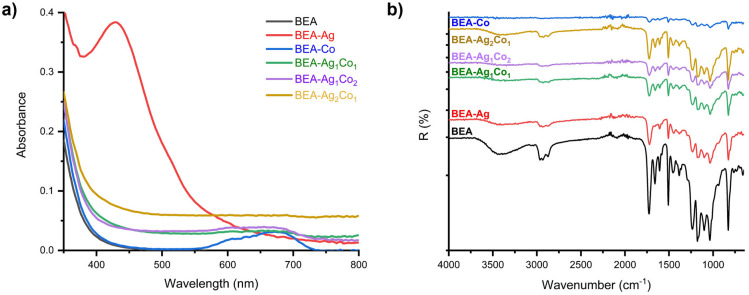
(**a**) Absorption and (**b**) reflection (%) spectra of nanocomposite films prepared by coating EA/DEGEEA-based formulations onto glass substrates.

A mixture of EA and DEGEEA was selected to produce a highly versatile hybrid matrix for photopolymerization and simultaneous photoreduction of metal salts. With its relatively high viscosity and multifunctional reactive sites, EA forms a rigid, densely cross-linked network upon UV irradiation. On the other hand, DEGEEA acts in the formulation as a low-viscosity, monofunctional reactive diluent that increases the mobility of radicals and ionic species, which facilitates the homogeneous distribution of metal salts and improves overall film-forming properties. The color changes after curing the formulations listed in Table S1 are visually presented in Figure 1 and Figure S2. In fact, the metal salt ratio in the formulations was varied, but no significant differences were observed for their visual appearance on the glass substrate. Additionally, as shown in Figure 2, UV–Vis absorption and reflectance (%R) spectra were recorded simultaneously using a UV–Vis spectrophotometer and an FT-IR spectrophotometer, respectively.

As shown in Figure S1a–f, the UV–Vis absorption profiles of the formulations were first recorded in ethanol, and a similar approach was subsequently applied to formulations consisting of an epoxy diacrylate/DEGEEA monomer mixture, photoinitiator, and Ag-Co precursors. UV–Vis spectra were recorded for each formulation coated onto a glass substrate before and after irradiation, as a recording of the surface plasmon resonance (SPR) band is critical for understanding nanoparticle formation, size, and morphology.

The quantitative particle size analysis derived from SEM images was informative for the average sizes of NPs. For Ag-containing systems, a characteristic and relatively sharp SPR band centered around 430 nm was observed (average size ~200 nm), similar to that detected in the solvent system, confirming the in situ formation of metallic Ag NPs. When Co was introduced into the acrylate formulation (BEA-Co), the cured film exhibited a broad and low-intensity absorption band in the 600–700 nm region, consistent with the formation of cobalt oxide species (average size ~43 nm) rather than a classical plasmonic response. Changing the Ag/Co ratio, a similar but weaker Co-related band was observed for the BEA-Ag_1_Co_2_ nanocomposite compared to BEA-Co.

These results demonstrate that the Ag:Co ratio plays an important role in NP nucleation and growth. When the Ag content was held constant, and the Co content increased from 1 to 2, only minor changes in particle size were estimated (~300 and 316 nm, as shown in Figure S3 and Table S2).

However, doubling the Ag content led to a pronounced reduction in the average size of the hybrid NPs, indicating that Ag dominates the nucleation process and restricts excessive particle growth. In addition to compositional effects, the optical properties are strongly influenced by NP size. The results clearly confirm that the Ag:Co ratio governs nanoparticle nucleation and growth, thereby controlling the size-dependent plasmonic properties of the resulting hybrid nanocomposites.

The reflectance spectra of UV–cured nanocomposite films showed differences depending on the metal ion ratio. FT-IR reflection spectra of the pristine BEA polymer and nanocomposite coatings showed that the in situ formation of Ag, Co_3_O_4_, and Ag/Co_3_O_4_ NPs did not lead to significant chemical changes in the epoxy acrylate matrix. All samples exhibited characteristic absorption bands of the BEA polymer, including C=O stretching (~1720 cm^−1^), C–O–C stretching vibrations (1000–1300 cm^−1^), and aliphatic C–H stretching bands (~2850–2950 cm^−1^), confirming the preservation of the polymer backbone after in situ NP formation. The absence of new absorption bands indicates that the NPs are incorporated into the polymer network without forming covalent bonds. This is consistent with the photochemical in situ formation method.

### 3.2. Photopolymerization Kinetics of Nanocomposites by Photo-DSC

A series of EA/DEGEEA formulations was prepared with and without Ag^+^ and Co^2+^ salts in different ratios in order to determine the role of metal ion composition on the functional properties of the UV–cured nanocomposites. While the formulation BEA only contained the photoinitiator (0.1% *w*/*w* BAPO) and the EA/DEGEEA matrix, other formulations included various amounts of AgNO_3_ and CoCl_2_ (0.33–1% *w*/*w*). Hence, the prepared formulations provided information on radical generation, heat flow, and polymerization rate, helping to elucidate the interplay between photoreduction, nanoparticle growth, and acrylate crosslinking.

**Figure 3:**
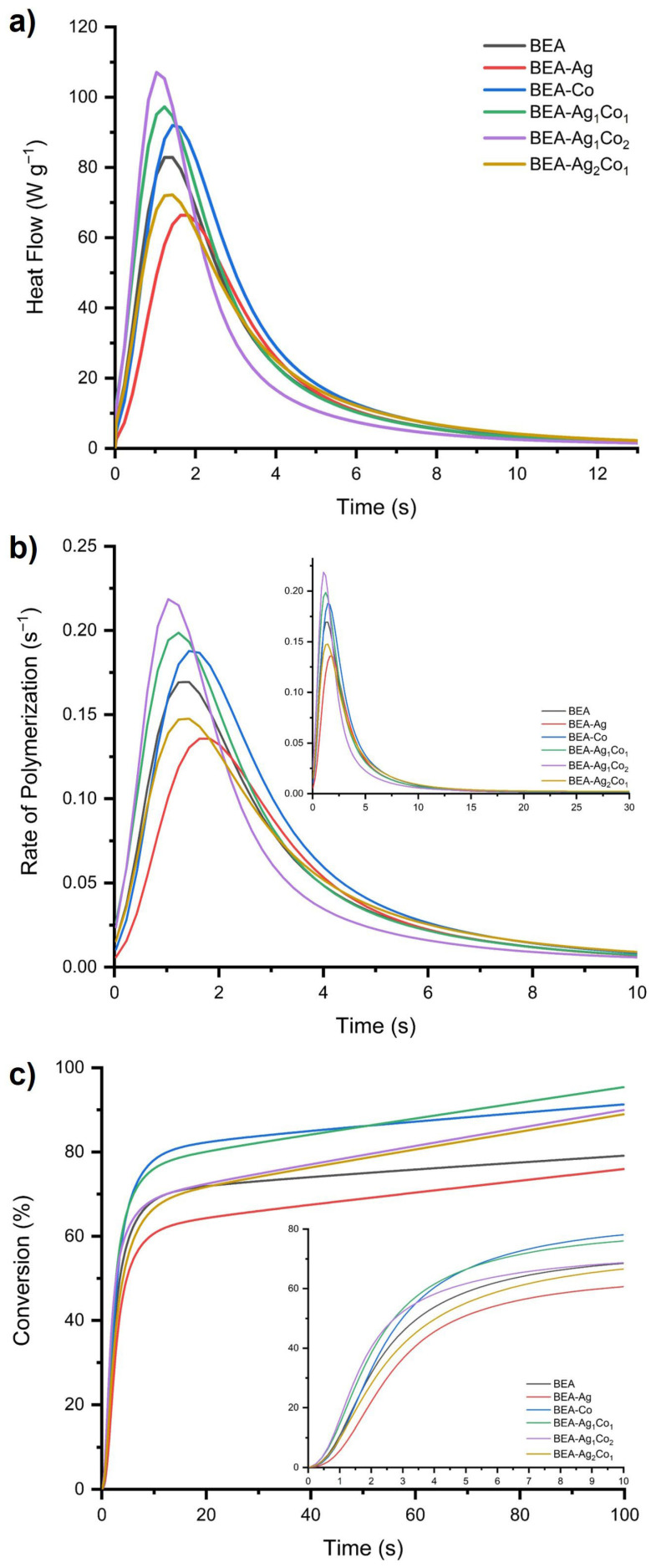
Photo-DSC results obtained for the EA/DEGEEA-based formulations: (**a**) heat flow, (**b**) rate of polymerization, (**c**) conversion (%, 100 s; in-set: 10 s).

Figure 3 presents the heat flow, polymerization rate, and conversion (%) curves obtained from these experiments, and the times corresponding to the maximum heat flow and polymerization rate, as well as the overall conversion values, are additionally summarized in Table 1. The polymer (BEA) exhibits a moderate heat-flow maximum and polymerization rate peak, whereas the introduction of Ag NPs results in a noticeable reduction in both parameters, possibly because of the SPR band of Ag NPs. Ag NPs compete with the absorption of the photoinitiator and affect initiation efficiency. In contrast, the Co-containing formulations (BEA-Co, BEA-Ag_1_Co_1_, and BEA-Ag_1_Co_2_) display substantially higher exothermic peak intensities and faster polymerization rates, demonstrating the strong catalytic role of Co species in enhancing radical generation. The most noticeable change was observed with BEA-Ag_1_Co_2_, which has the maximum heat flow (107.1 W·g^−1^) and the fastest R_p,max_ (0.219 s^−1^). This result suggests that adding more Co NPs speeds up the early curing stage better than consisting of more Ag NPs.

The conversion values at 10 s further support these kinetic trends. BEA-Co and BEA-Ag_1_Co_1_ reach the highest early conversions (78.1% and 76.0%, respectively), while BEA-Ag and BEA-Ag_2_Co_1_ exhibit comparatively slower conversion development. Among all formulations, BEA-Ag_1_Co_1_ shows the highest overall conversion (95.40%) in 100 s, surpassing both Co-only and Ag-rich systems. These results exhibited that the 1:1 ratio between Ag NPs and Co NPs is effective, increasing both the initial polymerization rate and overall conversion. The results confirm that Co NPs containing and particularly equimolar Ag/Co_3_O_4_ NPs hybrid systems significantly increase photocuring efficiency compared to both the pure polymer and Ag NPs containing formulations.

### 3.3. Thermal Decomposition of Nanocomposites

**Figure 4:**
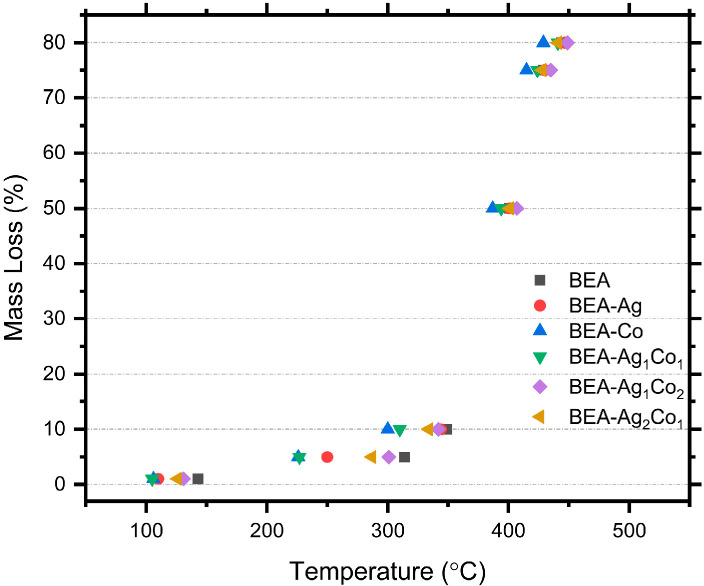
Mass loss (%) values of the EA/DEGEEA-based nanocomposite materials.

The thermal degradation profiles of the polymer (BEA) and prepared nanocomposites with in situ formed NPs, at various ratios of Ag/Co (BEA-Ag, BEA-Co, BEA-Ag_1_Co_1_, BEA-Ag_1_Co_2_, and BEA-Ag_2_Co_1_), displayed similar mass-loss trends (Figure 4 and Table 2). It was observed that in situ formed Ag and Ag/Co or Co NPs did not drastically alter the overall degradation mechanism of the EA matrix. All samples showed an initial low mass loss below approximately 150 °C, corresponding to the evaporation of residual solvents or unreacted monomers.

The main degradation occurred between 300 and 420 °C, reaching a mass loss of approximately 50–55%—a major weight loss, which is characteristic of the thermal breakdown of the acrylate crosslinked network. When Ag, Co, and various ratios of Ag/Co NPs were formed, no significant shift in the onset of degradation was observed, suggesting that the polymer network architecture remains dominant in determining thermal stability.

The differences in thermal decomposition of nanocomposites, containing different ratios of Ag/Co NPs hybrid systems and Co NPs, may be attributed to the distribution level of NPs in the polymer matrix, catalytic effects during polymer decomposition, or varying crosslink density in the system. A higher mass loss was found in formulations that had a higher amount of Co NPs at intermediate temperatures, possibly because of catalytic bond breakage caused by Co NPs.

Final char yields above 450 °C were similar in all measured samples, supporting that NPs formation occurred at low concentrations and did not significantly alter the final decomposition residue. Also, the results indicate that the nanocomposites retained the intrinsic thermal properties of the base polymer, with only minor differences that can be attributed to the metal ion composition and the presence of NPs.

### 3.4. Surface Properties and Crystal Structures of the Nanocoatings

The morphological characterization of the EA/DEGEEA nanocomposite materials was evaluated using SEM, while the crystalline phase and crystallite size characteristics of the same samples were analyzed by XRD in conjunction with the Scherrer method. Based on the combined assessment of the SEM and XRD results, the surface morphology, phase distribution, and nano-/micro-scale structural variations in the nanocomposites were examined.

**Figure 5:**
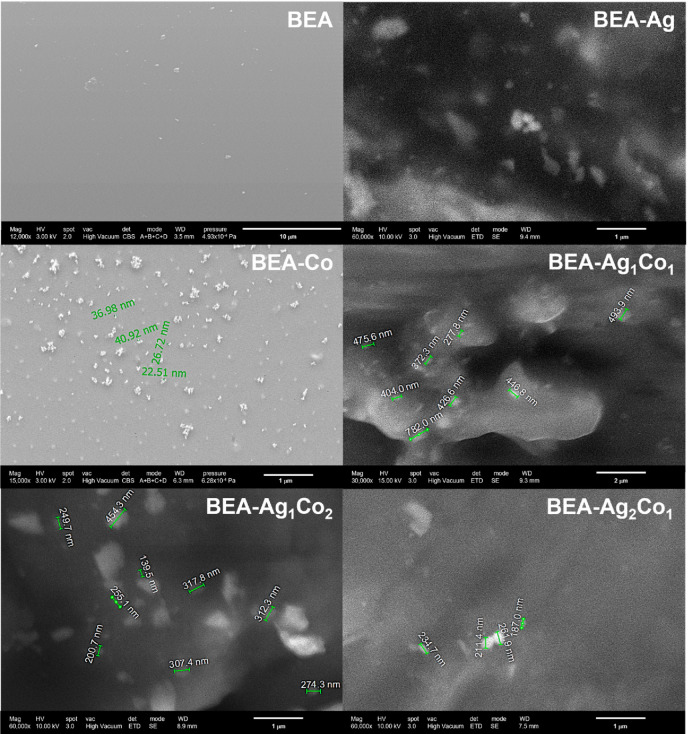
SEM images of the EA/DEGEEA-based nanocomposite materials.

SEM images of the samples are presented in Figure 5. Particle size analysis was performed using Fiji (ImageJ, 1.54p) software [47]. SEM images were calibrated using the scale bar and are provided in the Supporting Information (Figure S3 and Table S2). The polymer (BEA) exhibits a smooth and homogeneous morphology, confirming the amorphous structure of the polymer matrix. Among the samples, the BEA-Co nanocomposite exhibits the smallest average particle size, 42.3 nm. The formation of Co_3_O_4_ NPs due to slow growth and nucleation is more effectively restricted under the applied photochemical conditions.

In the case of Ag-containing nanocomposites, they display larger particle sizes (~200 nm), reflecting the well-known tendency of metallic Ag NPs to grow and coalesce during in situ photoreduction. The hybrid Ag/Co systems (BEA-Ag_1_Co_1_ and BEA-Ag_1_Co_2_) display the largest average particle sizes (~300–316 nm), indicating that the simultaneous presence of Ag and Co species promotes particle aggregation and/or heterogeneous nucleation, leading to enhanced particle growth. Interestingly, increasing the Ag content in the hybrid system (BEA-Ag_2_Co_1_) results in a reduction in the average particle size to 200 nm, comparable to that of the Ag-only system. This observation suggests that Ag plays a dominant role in controlling NP nucleation and growth in Ag-rich formulations.

These small and relatively well-dispersed NPs indicate that in situ photochemical formation of metallic NPs within the coatings was successfully achieved. The more uniformly distributed particles in the samples containing Co NPs are consistent with the formation of the Co_3_O_4_ spinel phase, as indicated by the XRD results.

A distinct morphological differentiation was observed in the hybrid NPs (BEA-Ag_1_Co_1_, BEA-Ag_1_Co_2_, and BEA-Ag_2_Co_1_), with larger aggregates (200–500 nm). The fact that these clusters do not form a single monolithic crystal is also supported by the crystallite size values obtained from XRD. Co ions may modify the surface redox environment, thereby accelerating the oxidation of Ag^0^ to Ag_2_O under ambient conditions.

**Figure 6:**
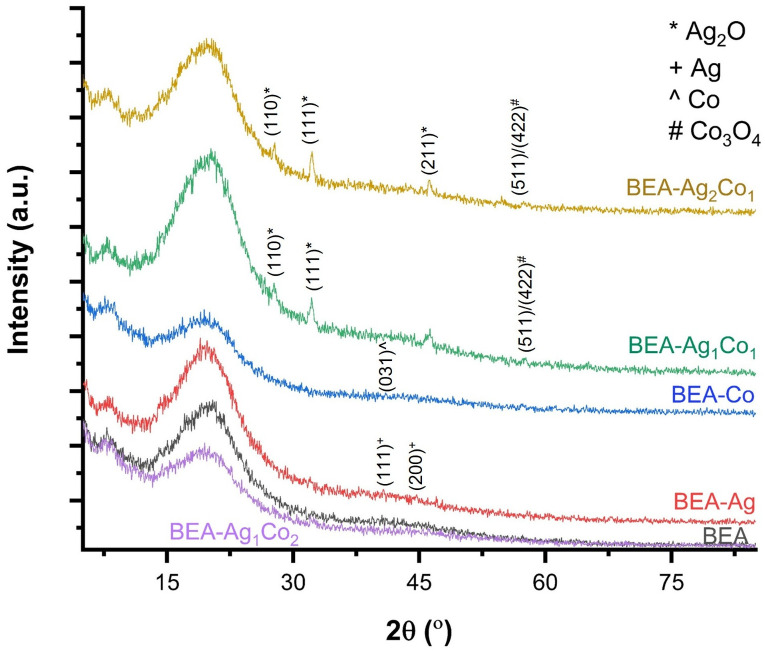
XRD diffraction patterns of EA/DEGEEA-based nanocomposite materials.

When the X-ray diffraction patterns of the samples are examined in Figure 6, it is seen that the polymer matrix exhibits a broad amorphous structure around 2θ ≈ 19–20° in all samples. The development of the amorphous structure of the BEA film is positively affected by the addition of Ag NPs, while it is weakened by the addition of only Co and the hybrid content with excess Co (BEA-Ag_1_Co_2_). In the samples containing metallic NPs, characteristic cubic Ag peaks (111), (200) and peaks corresponding to the complete transformation of Co into the Co_3_O_4_ spinel phase (JCPDS 42-1467) (i.e., (220), (311), (400), and (511)/(422)) were clearly recorded in the sample, in accordance with JCPDS, No. 04-0783. The formation of Ag_2_O (JCPDS, No. 76-1393) NPs in the structure was clearly observed upon the addition of hybrid BEA-Ag_2_Co_1_ and BEA-Ag_1_Co_1_. It was determined that increasing the amount of Co content in the hybrid Ag/Co system (BEA-Ag_1_Co_2_) suppresses the formation of continuous metallic Ag conductive pathways, thereby limiting the enhancement in dielectric permittivity.

Crystallite size calculations were performed using the Debye–Scherrer Equation (2) for the most intense peaks belonging to the Ag NPs, Ag_2_O, and Co_3_O_4_ phases.

(2) $$D=\frac{K\lambda }{\beta \cos\theta }\;\;\;$$

where K = 0.9 is the shape factor, *λ* = 1.5406 Å (Cu Kα radiation), and β is the full width at half maximum (FWHM). It was calculated that the size of Ag NPs in the BEA-Ag nanocomposite film is between 56 and 98 nm, and the size of the Ag_2_O NPs in the BEA-Ag_2_Co_1_ nanocomposite is between 43 and 53 nm.

### 3.5. Dielectric Properties of the Prepared Nanocoatings

The dielectric properties of the EA/DEGEEA-based composite and nanocomposite samples were evaluated using impedance spectroscopy. Impedance spectroscopy is a suitable and important technique for determining the dielectric properties and AC conductivity of polymer materials. The complex dielectric constant of a material is defined by Equation (3):

(3) $$\varepsilon^{*}(\omega )\;=\;\varepsilon (\omega )\;-\;i\varepsilon^{\prime }\;(\omega )$$

Here ε(ω) denotes the real part of the dielectric constant (dielectric permittivity), $\varepsilon^{\prime }(\omega )$ represents the imaginary part (dielectric loss component). These quantities are calculated using the following expressions:

(4) $$\varepsilon =\frac{Cd}{\varepsilon_{o}A}$$

(5) $$\varepsilon^{\prime }=\frac{\sigma }{{\omega \varepsilon }_{o}}$$

In these equations, C is the measured capacitance, d is the sample thickness, A is the electrode contact area, σ is the ionic/AC conductivity of the material, ε_0_ is the dielectric permittivity of free space, and ω is the angular frequency (ω=2πf).

**Figure 7:**
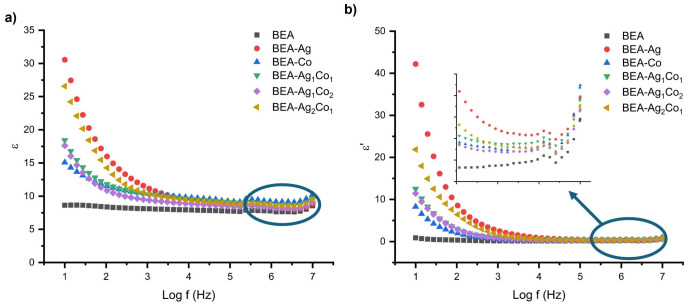
(**a**) Real and (**b**) imaginary parts of the permittivity of EA/DEGEEA-based composite/nanocomposite materials.

Frequency-dependent variations in the real and imaginary parts of the dielectric constant for EA-based composite and nanocomposite samples are presented in Figure 7a,b, respectively. For all materials, both ε and ε′ values increase with decreasing frequency. High dielectric permittivity at low frequencies is due to Maxwell–Wagner type space charge polarization caused by charge accumulation at conductor–insulator interfaces [48,49,50].

As shown in Figure 7a, the addition of metallic and metal/metal oxide NPs has a positive effect on the dielectric constant of the material, which leads to a general increase in the frequency region < 1 kHz. This increase is more pronounced in nanocomposites containing Ag NPs. In the low frequency region < 1 kHz, the highest dielectric constant value was obtained with the addition of 1% Ag, while a smaller increase was observed with the addition of 1% Co. The dielectric permittivity of the hybrid Ag/Co-containing nanocomposites lies between the values obtained for the BEA-Ag and BEA-Co systems and systematically increases with increasing Ag content. Furthermore, at higher frequencies (>10 kHz), the BEA-Co nanocomposite exhibits a higher dielectric constant compared to both the BEA composite and the BEA-Ag nanocomposite. The dielectric parameters of the nanocomposite materials at the selected frequencies are summarized in Table 3.

The reason why BEA films obtained by adding only Ag NPs have higher dielectric properties compared to films obtained by adding only Co NPs is that Ag NPs disperse at the interfaces without undergoing oxidation, as observed in the XRD patterns. The lower electrical conductivity of Co compared to Ag slows down the effective polarization. The limited enhancement in dielectric constant in the hybrid Ag/Co-containing formulations can be attributed to the crystallization of Co_3_O_4_ and Ag_2_O phases, as revealed by XRD (Figure 6), which disrupts the formation of continuous conductive pathways compared to metallic Ag NPs.

**Figure 8:**
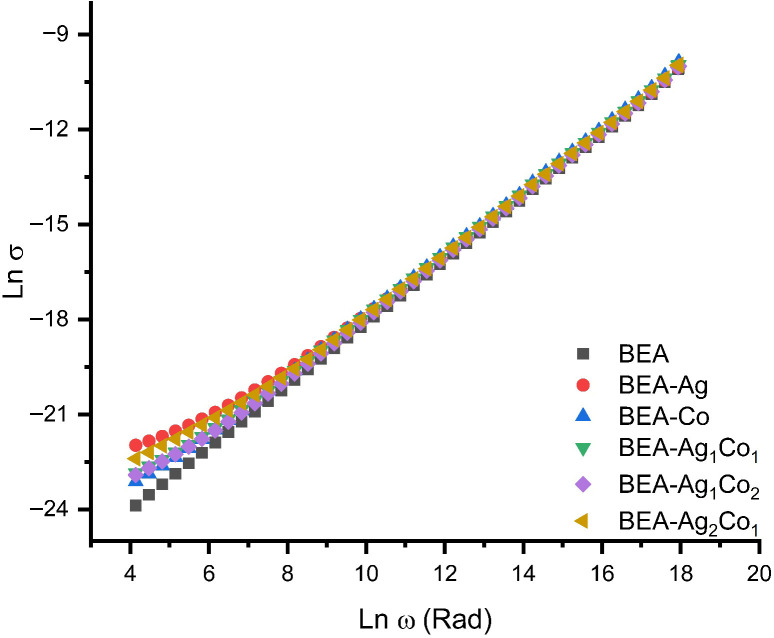
Frequency dependence of the AC conductivity of the EA/DEGEEA composite/nanocomposite films.

As shown in Figure 7b, the dielectric losses of nanocomposite films with added all-metallic and metal/metal oxide NPs are higher than those of the pure BEA film across the entire frequency range. The increase in dielectric loss with increasing dielectric constant is attributed to the maximum polarization of the dipole at low frequencies and minimum dipole polarization at higher frequencies [12,51]. The absence of effective dielectric loss at high frequencies with the addition of NPs may be related to the non-homogeneous distribution of the added NPs; this is supported by conductivity measurements. No NP effect was observed in the increased conductivity with increasing frequency (Figure 8).

AC-like and DC-like conductivities in a material correspond to different processes, and the total conductivity is given by Equation (6):

(6) $$\sigma (\omega )=\sigma_{\mathrm{AC}}(\omega )+\sigma_{\mathrm{DC}}$$

Here, $\sigma_{\mathrm{AC}}(\omega )$ represents the AC-like conductivity, $\sigma_{\mathrm{DC}}$ denotes the DC-like conductivity of the material, and σ(ω) is the total conductivity.

The AC conductivity is expressed by Equation (7):

(7) $$\sigma_{\mathrm{AC}}(\omega )=A\;\omega^{s}$$

where A is a constant dependent on temperature and s is the frequency exponent, taking values in the range 0 < s < 1. The frequency dependence of the AC electrical conductivity for the EA-based composite and nanocomposite materials is presented in Figure 8.

The electrical conductivity of a solid material composed of different regions increases with increasing frequency. At high frequencies, localized charge carrier movement is active in the conductive regions, while at low frequencies, charge transport is dependent on the presence of a continuous conductive region. The increase in the dielectric constant was determined to be proportional to the increase in the electrical conductivity of our material; this increase is more pronounced at low frequencies compared to the high frequencies [12,52].

It was determined that the dielectric constant increase at low frequencies was high in materials prepared with the addition of 1% Ag NPs, while the increase was relatively higher with the addition of only 1% Co NPs as the frequency range increased. The DC-like electrical conductivities of BEA, BEA-Ag 1%, and BEA-Co 1% nanocomposite films were calculated as 4.27 × 10^−11^, 2.87 × 10^−10^, and 9.09 × 10^−11^ S cm^−1^, respectively. While the DC-like conductivity increased 7-fold for 1% Ag with the addition of metallic NPs and approximately 2-fold with the addition of 1% Co, the addition of metal/metal oxide NPs allowed it to be controlled in the range of 2.87 × 10^−10^–9.09 × 10^−11^ S cm^−1^.

As can be seen from Figure 8, the AC-like conductivity of both Co and Ag 1% additives does not change compared to BEA. It was determined that the selected concentration was below the percolation threshold, and the situation was similar for both additives, and both showed similar frequency–conductivity behavior [49].

**Figure 9:**
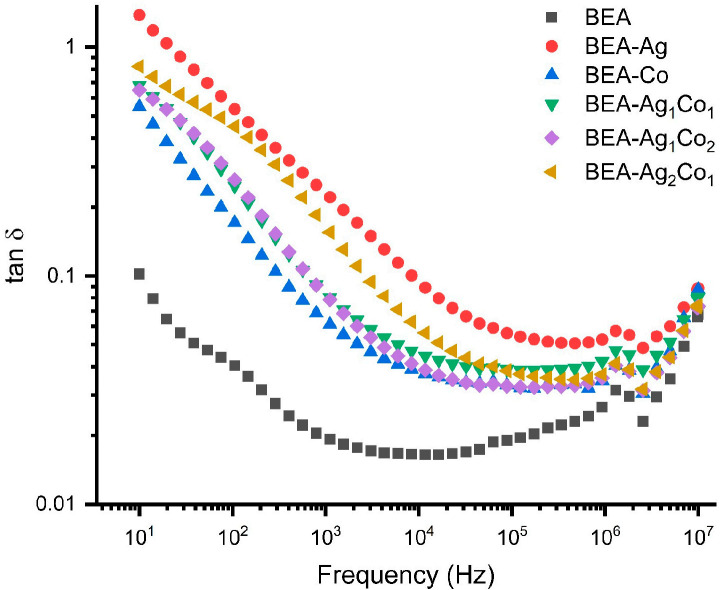
Frequency-dependent dielectric losses of the EA/DEGEEA-based composite/nanocomposite materials.

Figure 9 illustrates the frequency dependence of the dielectric loss factor (tan δ) for the EA/DEGEEA composite and nanoparticle-containing films. For all materials, tan δ values are relatively high in the low-frequency region and decrease significantly as the frequency increases. This behavior is related to electrode polarization and Maxwell–Wagner–Sillars type interface polarization with non-homogeneous deposition of charge carriers at the interfaces. As the frequency increases, the dipoles are not able to follow the rapidly oscillating electric field, which leads to a decrease in the dielectric loss. The slight increase in the high-frequency region is related to the increased AC conductivity and activation of localized charge carriers at higher frequencies. Among all the nanocomposites, BEA-Ag and hybrid Ag/Co nanocomposites exhibited higher tan δ values. This is consistent with the increased interface polarization and conductivity contributions introduced by the metallic NPs. In contrast, the blank (BEA) shows the lowest loss factor throughout the whole frequency range. This is related to the limited dipole interactions and the relatively lower concentration of mobile charge carriers within the system.

**Figure 10:**
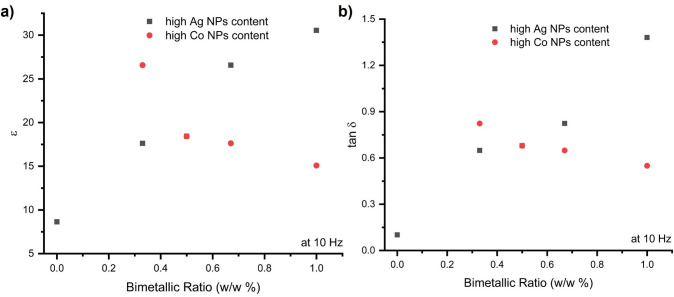
(**a**) Variation in the real part of the dielectric constant and (**b**) dielectric loss of the EA/DEGEEA-based composite/nanocomposite materials at 10 Hz.

Figure 10 shows the variation in dielectric permittivity and loss values at 10 Hz depending on the metal/metal oxide NPs addition ratio. Compared to the pristine BEA sample, all nanocomposite films exhibited an increase in dielectric permittivity. However, within the hybrid Ag–Co systems, increasing the Co content led to a gradual decrease in dielectric permittivity, whereas higher Ag content resulted in a more pronounced enhancement of dielectric permittivity.

This adjustable dielectric permittivity and dielectric loss factor indicate that nanocomposite thin films can be used in the production of layered capacitors. It would be possible to minimize dielectric loss while creating a capacitor with the desired dielectric permittivity. Furthermore, using it as an outer layer with low dielectric loss would increase the breakdown resistance at high voltages. In addition, it may also have application areas in capacitive sensor applications as adjustable coatings to reduce signal-to-noise interference.

### 3.6. Magnetic Properties of the Prepared Nanocoatings

**Figure 11:**
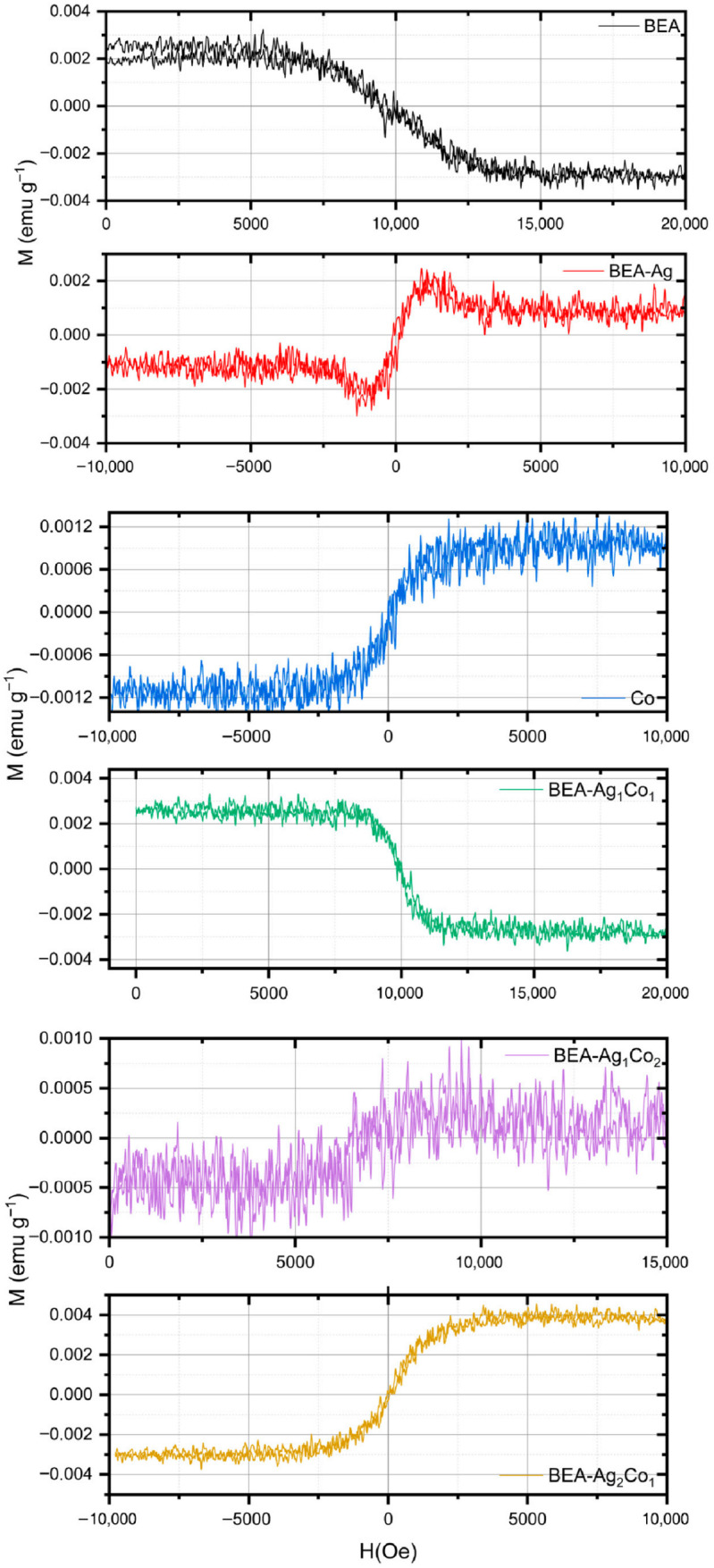
Hysteresis loops of the nanocomposite films containing Ag and/or Co NPs.

The magnetic properties of the nanocomposites containing Ag and/or Co NPs were analyzed using the hysteresis curves as presented in Figure 11. This figure shows the magnetization (M) response under an applied magnetic field (H). Some key magnetic parameters, such as saturation magnetization (M_s_), remanent magnetization (M_r_), and coercive field (H_c_), were determined from the hysteresis curves.

The magnetization level of the BEA empty matrix is very low (M_s_ ≈ 0.003 emu g^−1^; M_r_ ≈ 0; H_c_ ≈ 0 Oe), and the hysteresis curve does not form a closed loop. This indicates that BEA is nonmagnetic. The BEA-Ag formulation material does not show a significant magnetic response. The M_s_ value is low (≈0.0022 emu g^−1^), and the M_r_ value is almost zero; this is consistent with the non-magnetic nature of Ag NPs, supported by the fact that Ag NPs are in a metallic form in the XRD patterns.

The addition of only Co NPs (BEA-Co) imparts very weak ferromagnetic behavior. The hysteresis loop is well-defined and symmetrical. The estimated M_r_ (≈0.0012 emu g^−1^) and H_c_ (approximately 100–150 Oe) values confirm that the material retains its magnetization even after the external field is removed.

The BEA-Ag_2_Co_1_ formulation shows the highest magnetic response among all the other samples. Both of the M_s_ values were found to be significantly increased by up to 2-fold compared to the BEA-Ag and BEA-Co nanocomposites. These findings indicate that the transformation of Ag NPs into the Ag_2_O structures, as determined in the XRD patterns, supports the magnetic response in a way that enhances superparamagnetic properties.

## 4. Conclusions

This study reports a novel, reproducible, and facile photochemical technique to prepare in situ metallic and hybrid metal/metal oxide nanocomposites. The SPR bands of the Ag NPs, Co NPs, and hybrid Ag@Co NPs were confirmed by UV–Vis absorption spectroscopy technique, and the reflectance of the nanocomposites was also recorded by FT-IR spectroscopy. Thermal decomposition of pure polymer and nanocomposites was followed by TGA. TGA results indicate that the formation of NPs does not fundamentally alter the thermal degradation mechanism of the EA/DEGEEA polymer matrix. The similarity of main-chain degradation temperatures across all samples confirms that Ag and Co NPs have only a minor impact on the intrinsic thermal stability of the polymer backbone. Photopolymerization kinetics were followed by Photo-DSC by means of heat flow, the rate of polymerization, and conversion values. The results demonstrate that Co-based nanocomposites, particularly the hybrid BEA-Ag_1_Co_2_ formulation, provide the highest photocuring efficiency, exhibiting the fastest initial reaction, highest heat-flow intensity, increased polymerization rate, and higher monomer conversion. These findings confirm that metallic NPs play a decisive kinetic role in modulating the photopolymerization behavior of EA/DEGEEA systems, principally through their impact on radical generation and chain-initiation dynamics.

Formation of metallic and hybrid metal/metal-oxide NPs within BEA nanocomposite films results in the formation of metallic Ag NPs similar to those in Ag-based systems, leading to a 7-fold increase in dielectric permittivity. In comparison, the formulation consisting of Co_3_O_4_ NPs (1% Co precursor) produces a more moderate enhancement in dielectric permittivity, attributed to the lower electrical conductivity of Co and the formation of weaker conductive networks. The formation of hybrid Ag/Co NPs enables precise tuning of dielectric permittivity over a 2–7-fold range. The controllable dielectric increase in the low-frequency region in EA films is noteworthy for their potential use in energy storage. Tunable dielectric properties arising from the in situ photochemical formation of metallic Ag and hybrid Ag/Co_3_O_4_ NPs enable new application areas, particularly in layered capacitors and sensor devices.

## Figures and Tables

**Table 1:** Maximum heat flow, maximum rate of polymerization, and conversion values obtained from the photo-DSC analyses.

Formulation	t_max,heat_ (s)	q_max_ (W g^−1^)	t_max,rate_ (s)	R_max,polym._ (s^−1^)	Conversion (%)	
					10 s	100 s
BEA	1.43	82.9	1.43	0.169	68.5	79.1
BEA-Ag	1.63	66.4	1.63	0.136	60.7	75.9
BEA-Co	1.43	92	1.43	0.188	78.1	91.3
BEA-Ag_1_-Co_1_	1.23	97.3	1.23	0.199	76	95.4
BEA-Ag_1_-Co_2_	1.03	107.1	1.03	0.219	68.7	89.9
BEA-Ag_2_-Co_1_	1.43	72.2	1.43	0.148	66.6	88.9

**Table 2:** Summary of the mass losses (%) obtained from the DT-TGA results.

**Formulation**	**Mass Loss (%)**				
	**1**	**10**	**50**	**80**	**Temperature (°C)**
BEA	143	349	401	444	
BEA-Ag	110	344	401	447	
BEA-Co	106	300	387	429	
BEA-Ag_1_Co_1_	105	310	394	441	
BEA-Ag_1_Co_2_	131	342	407	449	
BEA-Ag_2_Co_1_	126	334	401	441	

**Table 3:** Dielectric parameters of the nanocomposite films at different frequencies.

Frequency (Hz)	ε		
	BEA	BEA-Ag	BEA-Co
10	8.6	30.6	15.1
10^2^	8.3	16	11.5
10^3^	8.1	11.2	10.3
10^4^	7.9	9.4	9.8
10^5^	7.7	8.6	9.2
10^7^	8.6	8.9	10.3

## Data Availability

The original contributions presented in this study are included in the article/Supplementary Materials. Further inquiries can be directed to the corresponding author.

## Supplementary Materials

The following supporting information can be downloaded at: https://www.mdpi.com/article/10.3390/nano16020143/s1, Figure S1: Changes in the UV–Visible absorption spectra of the formulations as a function of irradiation time in ethanol: (a) BAPO, (b) B–Ag, (c) B–Co, (d) B–Ag_1_Co_1_, (e) B–Ag_1_Co_2_, and (f) B–Ag_2_Co_1_, showing the time-dependent formation of SPR bands.; Table S1: Composition of the prepared nanocomposite materials; Figure S2: Changes observed during the curing process of EA/DEGEEA-based nanocomposite films coated onto glass substrates: (a) BEA, (b) BEA-Ag, (c) BEA-Co, (d) BEA-Ag_1_Co_1_, (e) BEA-Ag_1_Co_2_, (f) BEA-Ag_2_Co_1_. Figure S3: Particle size distribution histograms of NPs obtained from calibrated SEM images (a) BEA-Ag, (b) BEA-Co, (c) BEA-Ag_1_Co_1_, (d) BEA-Ag_1_Co_2_, (e) BEA-Ag_2_Co_1_. Table S2: Average particle size values of NPs calculated from SEM images.
