# Supplementary material for: Tuning Ag/Co Metal Ion Composition to Control In Situ Nanoparticle Formation, Photochemical Behavior, and Magnetic–Dielectric Properties of UV–Cured Epoxy Diacrylate Nanocomposites
Source: Nanomaterials (Basel). 2026 Jan 21;16(2):143. doi: 10.3390/nano16020143 (PMC12844846; doi:10.3390/nano16020143)
Supplement: Supplementary file 1 [file nanomaterials-16-00143-s001.zip › nanomaterials-4103286-supplementary.pdf]

# - SUPPORTING INFORMATION -

Tuning Ag/Co Metal–Ion Composition to Control in Situ Nanoparticle Formation, Photochemical Behavior, and Magnetic–Dielectric Properties in UV-Cured Epoxy Diacrylate Nanocomposites

Gonul S. Batibay[1,2], Sureyya Aydin Yuksel[3], Meral Aydin[1], Nergis Arsu[1]

[1]*Department of Chemistry, Yıldız Technical University, Istanbul 34220, Turkey*
[2]*Research Laboratories Application and Research Center (ALUAM), Turkish-German University, Istanbul 34820, Turkey*
[3]*Department of Physics, Yıldız Technical University, Istanbul 34220, Turkey*
**Correspondence: narsu@yildiz.edu.tr*

- **UV-Induced Optical Changes during in-Situ NP Formation**

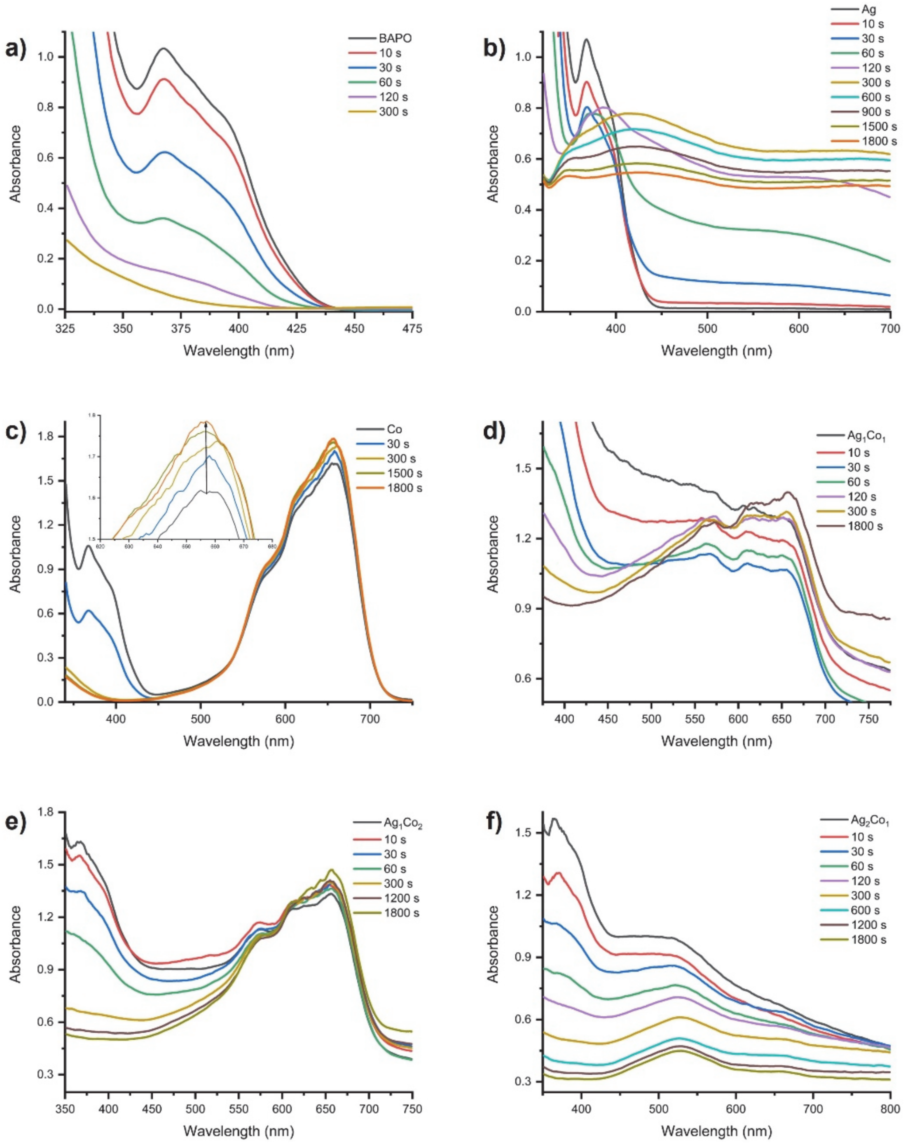

Figure S1. Changes in the UV–Visible absorption spectra of the formulations as a function of irradiation time in ethanol: **(a)** BAPO, **(b)** B–Ag, **(c)** B–Co, **(d)** B–Ag_1Co_1, **(e)** B–Ag_1Co_2, and **(f)** B–Ag_2Co_1, showing the time-dependent formation of SPR bands.

Time-dependent UV-Visible absorption spectra shows changes in optical properties during UV irradiation, which corresponds to the in-situ formation of nanoparticles. In the Figure 1a, photolysis of the photoinitiator BAPO was performed, and a rapid decrease in UV absorbance was observed. In **Figure S1b**, in the Ag-containing system **(b)**, the gradual emergence and stabilization of a surface plasmon resonance (SPR) band around 400-430 nm is typical for the formation of metallic Ag NPs.

In the Co-containing formulation **(c)**, no distinct SPR band is observed; instead, a broad visible absorption band centered around 600-700 nm develops, consistent with the formation of cobalt oxide (Co_3O_4) instead of metallic cobalt. In hybrid Ag-Co systems **(d-f)**, both Ag-related SPR properties and Co_3O_4-related broad visible absorption bands were detected. The shape and intensity of the SPR bands vary depending on the ratio of precursors and the irradiation time.

In general, these spectra confirm irradiation-directed in-situ nanoparticle formation and demonstrate tunable optical behavior through controlled Ag/Co composition.

Table S1. Composition of the prepared nanocomposite materials

**Formulation**	**Composition (%w/w)**				
	**BAPO**	**$AgNO_3$**	**$CoCl_2$**	**EA**	**DEGEEA**
**BEA**	0.5	-	-	69.65	29.85
**BEA-Ag**	0.5	1	-	68.95	29.55
**BEA-Co**	0.5	-	1	68.95	29.55
**BEA-Ag_1Co_1**	0.5	0.5	0.5	68.95	29.55
**BEA-Ag_1Co_2**	0.5	0.33	0.67	68.95	29.55
**BEA-Ag_2Co_1**	0.5	0.67	0.33	68.95	29.55

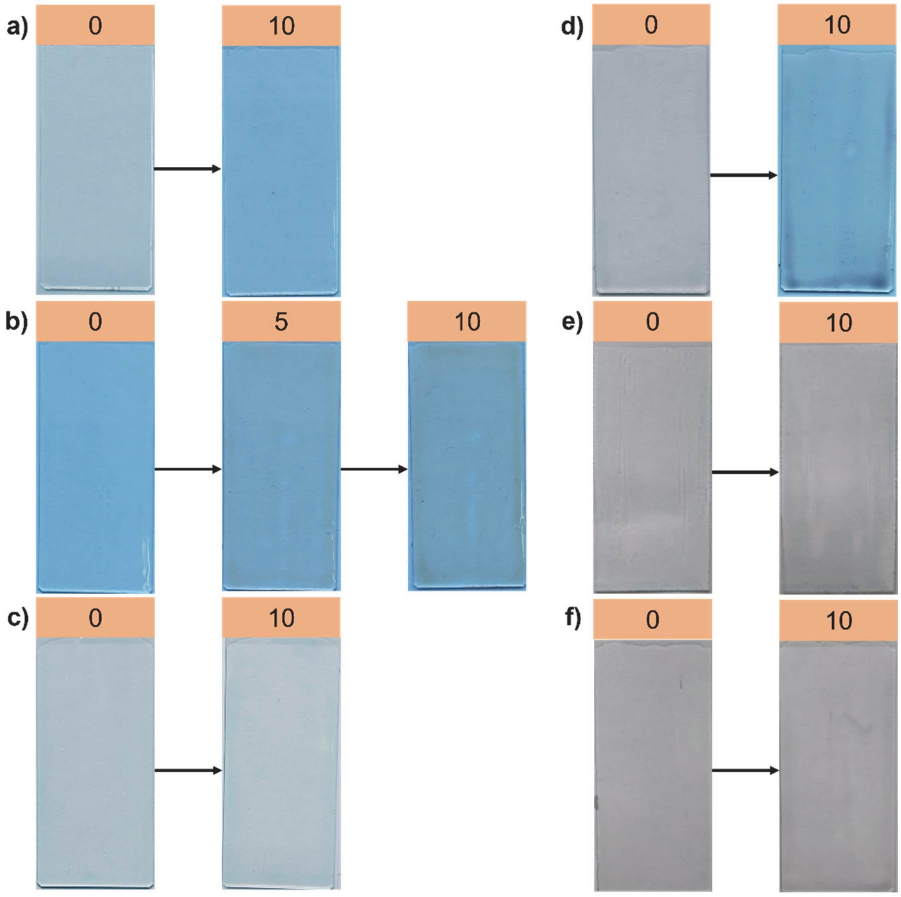

Figure S2. Changes observed during the curing process of EA/DEGEEA-based nanocomposite films coated onto glass substrates: **(a)** BEA, **(b)** BEA-Ag, **(c)** BEA-Co, **(d)** BEA-Ag_1Co_1, **(e)** BEA-Ag_1Co_2, **(f)** BEA-Ag_2Co_1

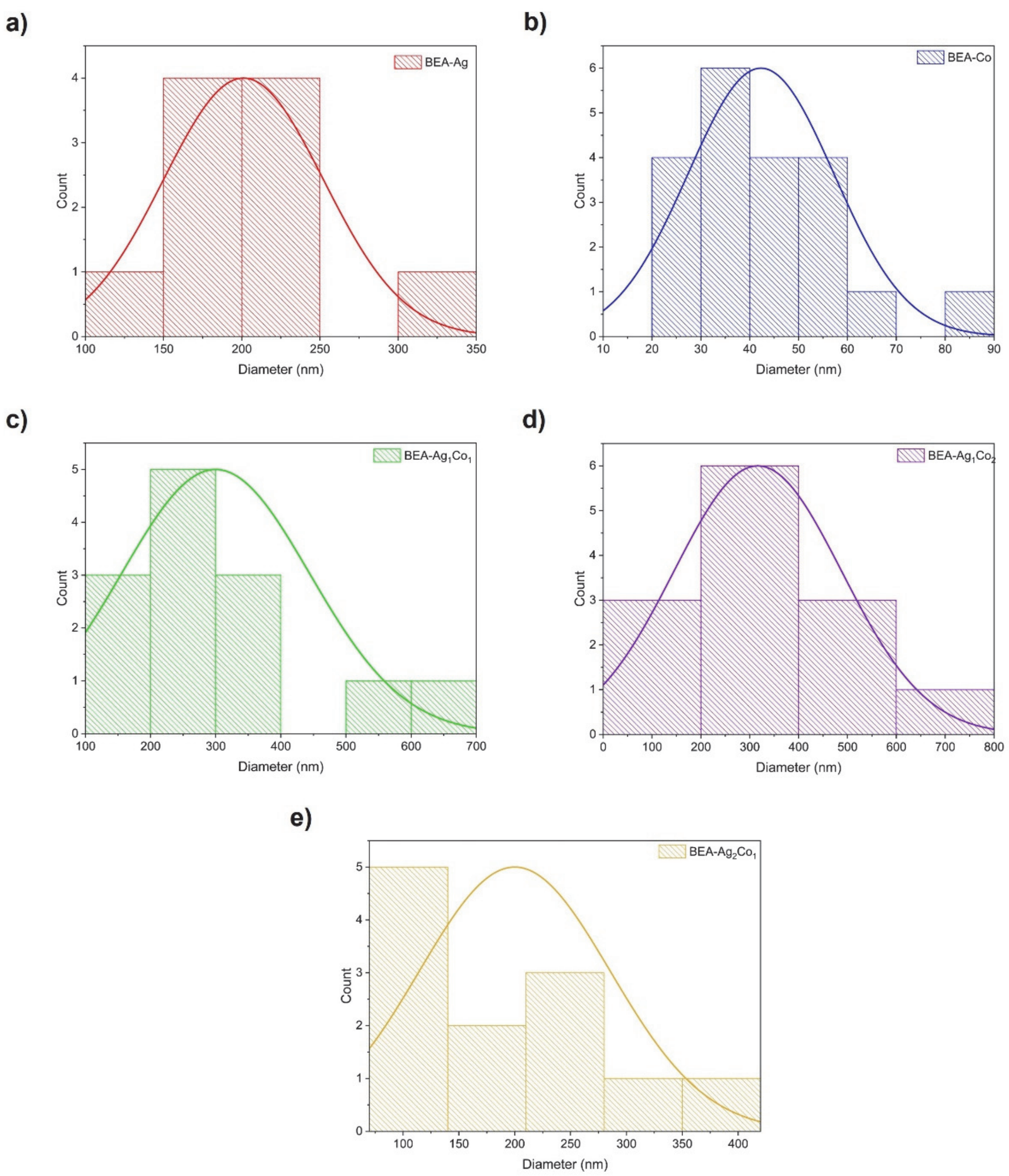

Figure S3. Particle size distribution histograms of NPs obtained from calibrated SEM images **a)** BEA-Ag, **b)** BEA-Co, **c)** BEA-Ag_1Co_1, **d)** BEA-Ag_1Co_2, **e)** BEA-Ag_2Co_1

Table S2. Average particle size values of NPs calculated from SEM images

Nanocomposite Name	Average of particle size (nm)
**BEA-Ag**	201.1
**BEA-Co**	42.3
**BEA-Ag_1Co_1**	299.8
**BEA-Ag_1Co_2**	316.4
**BEA-Ag_2Co_1**	200.0
